# Edible and Medicinal Progress of *Cryptotympana atrata* (Fabricius) in China

**DOI:** 10.3390/nu15194266

**Published:** 2023-10-05

**Authors:** Xingcheng Xie, Han Guo, Juan Liu, Junbao Wang, Huihui Li, Zhongyuan Deng

**Affiliations:** 1 School of Agricultural Sciences, Zhengzhou University, Zhengzhou 450001, China; xingchengxie@126.com; 2 State Key Laboratory for Biology of Plant Diseases and Insect Pests, Institute of Plant Protection, Chinese Academy of Agricultural Sciences, Beijing 100193, China; 3 School of Life Sciences, Zhengzhou University, Zhengzhou 450001, China; guohan0220@126.com (H.G.); junbaowang2021@163.com (J.W.); hhlapple2022@163.com (H.L.); 4 Beijing Center for Disease Prevention and Control, Beijing Key Laboratory of Diagnostic and Traceability Technologies for Food Poisoning, Beijing 100013, China; liujuan0538@foxmail.com; 5 State Key Laboratory of Stem Cell and Reproductive Biology, Institute of Zoology, Chinese Academy of Sciences, Beijing 100101, China

**Keywords:** *Cryptotympana atrata*, edible insect, medicinal insect, cicada slough, *Cordyceps cicadae*

## Abstract

As an important resource insect, the *Cryptotympana atrata* is widely distributed in the eastern and central parts of China. The cicada slough is one of the traditional crude drugs in East Asia, and the main component is polysaccharide, which has the functions of anti-convulsion, relieving asthma and improving lipid metabolism. The parasitoid fungus *Cordyceps cicadae,* which grows inside the cicada nymphs and forms the fruiting bodies on the surface of the host’s carcass, is also known as the “cicada flower” in China. The *Cordyceps cicadae* is another old, traditional Chinese medicine, which has been used as a tonic and medicine to nourish and regulate human immunity for centuries. For the further development and utilization of the golden cicada, this paper summarized the *C. atrata* from the aspects of their biological characteristics, distribution area, life cycle, history of edible and medicinal use, edible methods and nutritional compositions; emphatically introduced the edible and potential medicinal value of the *C. atrata*; and specifically expounded the research progress of its application. As one popular insect food, the prospects for the development of *C. atrata* have also been put forward, especially in artificial breeding technology, food safety risk assessment and medicinal value utilization.

## 1. Introduction

The *Cryptotympana atrata* is an extensively-used food and medicine resource insect in China, which has been widely recorded in medical works of the past dynasties. This insect is a member of the order Hemiptera of the Cicadidae family. The Cicadidae family from China comprises more than 200 species in 62 genera [1], but the cicada used as food and medicine is the *Cryptotympana atrata* in China. The nymph of this insect can be made into many delicious foods, while the cicada slough also has a variety of functions in medicine.

The cicada slough is a traditional Chinese medicine with many clinical purposes. To verify its therapeutic effects and safety, the accurate identification and authentication of this species are essential and critical. The morphological characteristics are usually used for distinguishing the *Cryptotympana atrata* from other cicadae, especially by identifying the differences of antennae, the ninth and tenth sternites in males and the number of hind tibial spines in the final instar nymph [2].

Recently, the exuviae of cicadas has also been used for molecular identification among different cicadae species. The *COI* gene sequence of the *Cryptotympana atrata* has been analyzed, and the markers for the sequence-characterized amplified region have been successfully applied to the rapid identification of the cicada exuviae [3]. By the comparative analyses of the leg morphology, ultrastructure and mitochondrial DNA sequences of exuviae from four dominant cicada species including the *Cryptotympana atrata*, the results have also indicated that the leg morphological and exuvial molecular characteristics can possibly be highly reliable identification markers [4].

This paper describes the edible and medicinal value of the *Cryptotympana atrata* and its development prospects for further development and utilization. The specific areas for the utilization of this insect, such as insect food, health products, medicine and artificial breeding, will be expanded.

## 2. A Brief Introduction to the *Cryptotympana atrata*

### 2.1. Morphological Characteristics

**Figure 1:**
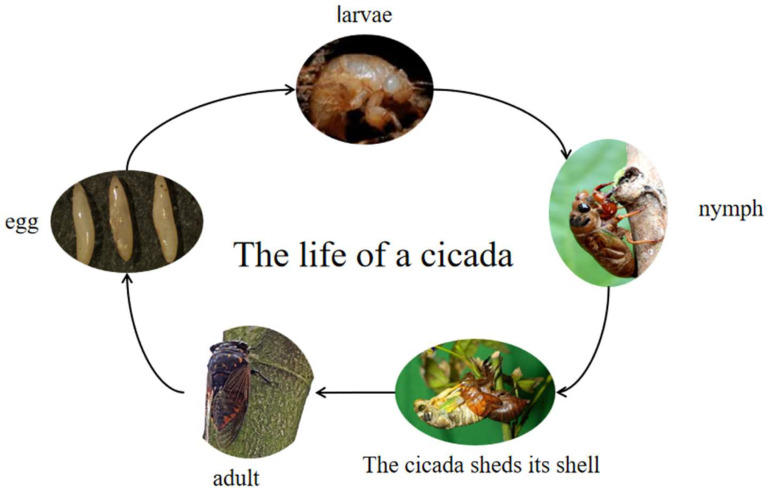
Life cycle of the *Cryptotympana atrata* [5].

The development mode of the *Cryptotympana atrata* is an incomplete metamorphosis, including three stages in its life: egg, nymph and adult (Figure 1) [5]. The newly laid egg is milky white or light yellow, glossy and fusiform. The whole body of the first instar nymph is milky white with red compound eyes, and the teeth of the first foot are slightly reddish [5,6]. The body weights of the first and second instar nymph are about 1 g and 3 g, respectively. After the third instar, wing buds appear, and the pigment gradually deepens to a grayish brown color. In the fifth instar nymph, the whole body is yellowish brown, and the shape is slightly like the adult. The fifth instar nymph has the characteristics of developed and opaque wing buds, the forehead and body are densely packed with brown setae and there is a developed forefoot of the fossorial foot [5]. Nymphs have a variety of common names, such as: golden cicada, climbing black cicada, cicada monkey and climbing fork, and different areas have different common names. 

Generally, the body of a male adult is black–brown or black and glossy with golden brown hair. The fore edge of the head is wider with the central depression, and the mouthparts are sucking. The three monocular eyes are located between the large and prominent compound eyes, and are arranged in a triangular shape. Both sides of the pronotum are slightly enlarged, and the mesonotum is broad, with a yellow–brown “x”-shaped eminence in the center. The fore and hind wing membranes are transparent, but the wings are very hard [5].

### 2.2. Living Habit

The *Cryptotympana atrata* ranges from Korea to China and the northern part of Southeast Asia, which occurs in many areas of China. It spawns in July and August every year. In late July, the female adult begins to lay eggs after mating, and uses the ovipositor to pick up the bark of the branch tip and pierce a number of sloping egg chambers 0.5–1.0 cm deep on the branch. Each egg chamber produces 6–8 fertilized eggs [3]. *Cryptotympana atrata* eggs have the characteristics of diapause and start to hatch in May or June after overwintering. The nymphs fall to the ground, start to search for an area with high humidity and soft soil and dig holes with their forelimbs to enter the soil. After entering, they obtain nutrition by sucking the sap of plant roots for continuous growth and development [5]. Nymphs generally live in the soil for 3–5 years, or even longer, until the final instars of the larvae emerge from the ground, climb on nearby weeds and trees and finally stay on the branches, leaves or bark in order to molt into adults. The behaviors of mating and oviposition begin at 20 days after emergence, with the ovipositing peak from late June to late August [7].

### 2.3. Regional Distribution

**Figure 2:**
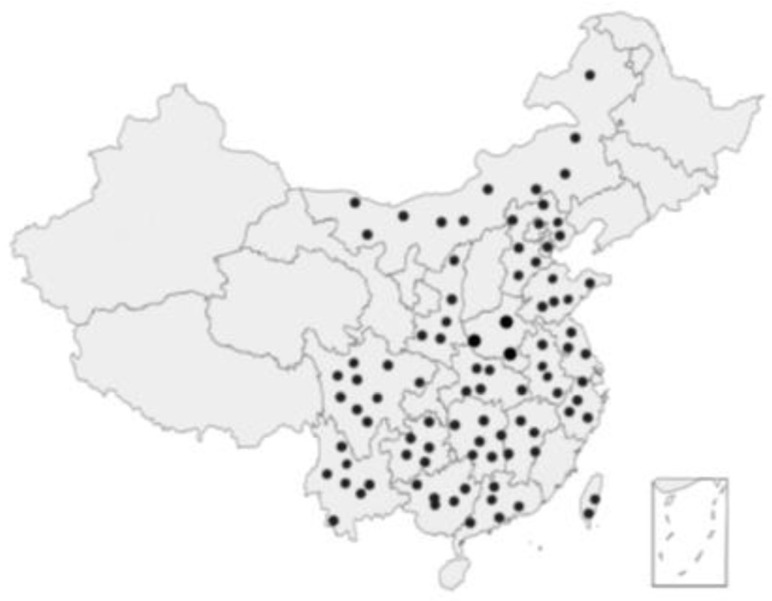
Distribution of the *Cryptotympana atrata* in China [9,10].

The *Cryptotympana atrata* mainly occurs in temperate and tropical regions. The main distribution areas of the *Cryptotympana atrata* in China are in Western Henan and the plains, foothills and low hills along the Yellow River and Yiluo River, away from the villages and towns [8]. The *Cryptotympana atrata* is widely distributed in many provinces including Shandong, Henan, Beijing, Nei Mongol, Tianjin, Hebei, Shaanxi, Shanghai, Jiangxi, Taiwan, Hunan, Guangdong, Jiangsu, Yunnan, Guizhou, Hubei, Anhui, Zhejiang and Sichuan (Figure 2) [1,9,10]. In particular, this insect occurs in areas with loose soil and more trees [8]. The *Cryptotympana atrata* can stay underground for three years or more. But for the cold zone with low temperatures in winter, the nymphs can hardly survive. For example, in Northwest China, where the differences between day and night temperatures are large with low precipitation, there are very few cicadae. In grassland areas, there are also very few cicadae because of the sparse trees.

## 3. The Edible History of the *Cryptotympana atrata* in China

Chinese people have a long history of feeding on the *Cryptotympana atrata*. The earliest history of eating cicadae was recorded in *The Book of Rites* written in 300 BC [1]. Confucian scholars in the Han Dynasty once said “Cicadae are edible insects, which can be eaten by all people from the emperors down to the lowest of the common people”. During the Three Kingdoms Period, the Jin Dynasty and the Southern and Northern Dynasties, people’s interest in the *Cryptotympana atrata* reached its peak, and a variety of ways to eat cicadae were also invented. According to the records, there were four main methods for the ancients to eat the *Cryptotympana atrata*, including baking, steaming, blanching and making meat broth [1]. Another conventional method was food preservation. Cicadae had a very short life span of just a few months. Thus, the ancients made dried meat from captured cicadae and kept it to extend the shelf life. These findings suggest that the ancients were very enthusiastic about eating the *Cryptotympana atrata*.

Not only in ancient times, but also today, eating cicadae is still one of the traditional food cultures for Chinese people [11]. Because cicadae are good at flying, their chest muscles are as developed as the chest muscles of birds, and their muscle fibers are delicate and delicious. There are many ways to eat them, such as in the form of dishes, processing them into candied fruits and condiments, as well as grinding them into powder for further processing into a variety of food mixed with flour. 

In most provinces of China, the *Cryptotympana atrata* is served in the form of dishes. In Guangdong and Hunan, the *Cryptotympana atrata* is stewed in plain water, while it is fried in Yunnan and Henan. Moreover, the *Cryptotympana atrata* can also be processed into candied fruit and flavoring. The Dai and Bulang nationalities living in Yunnan enjoy making cicadae into meat sauce.

In addition to the traditional dishes and primary products developed with cicadae as the main raw material, high and new technology such as bioengineering has greatly promoted the progress of producing cicada protein powder and amino acid oral liquid [12]. Insect protein food has been increasingly accepted by people recently. The extracted insect protein can be used as a natural health food additive in a variety of foods such as bread, biscuits, pastries and tonic drinks. Furthermore, insect health food is also emerging, such as the silkworm pupa processed by the fermentation method, silkworm pupa bean paste, silkworm pupa bread, etc. Therefore, there are broad prospects for the development of the *Cryptotympana atrata* to be healthcare products. 

Meanwhile, as an insect, the *Cryptotympana atrata* has certain fungi and parasites in its body, such as *Beauveria* and *Metarhizium*. It must be cooked at high temperatures, so that the parasites and fungi are completely killed. However, this insect food is not suitable for everyone. People with protein allergies and poor kidney function should not eat it. Scientists have found that the defense glands in the breast of the *Cryptotympana atrata* contain a hormone known to vertebrates, called corticosterone, the content of which reaches as high as 0.4 mg. Because corticosterone strongly disrupts the vertebrate potassium–sodium ratio, the amount should be strictly controlled to avoid breaking the balance of sodium and potassium in the human body [13].

**Figure 3:**
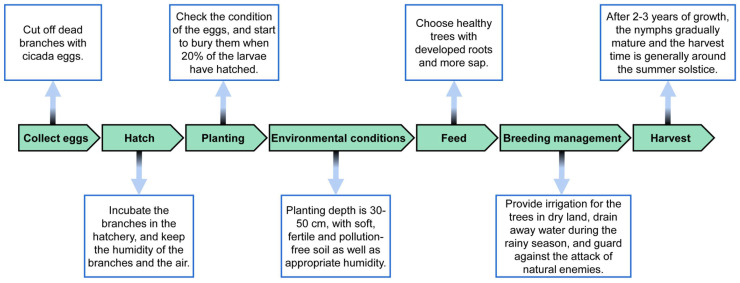
Technology process of artificial cicada rearing [14].

With the increasing demand of the *Cryptotympana atrata* for Chinese people, the artificial breeding of this insect has made great progress (Figure 3) [14]. In recent years, the cultivation technology of the *Cryptotympana atrata* under the forest has emerged, and there have been many successful cases such as under the willow, elm, poplar and mulberry [14,15]. Insect cultivation technology is inevitably becoming one type of promising green technology innovations with great economic benefits and a good development trend.

## 4. The Value of the *Cryptotympana atrata*

### 4.1. Nutritional Quality

The *Cryptotympana atrata* is rich in protein, and the ratio of protein to fat content (PFR) is >6 (Table 1) [16]. The crude protein content of the *Cryptotympana atrata* is 21.4 g/100 g measured by the Kjeldahl nitrogen determination method [17], which is 1.62 and 1.67 times the content in pork and egg, respectively. The protein content of the nymph reaches as high as 68.83 g/100 g in dry weight. The content of crude fat in the *Cryptotympana atrata* is 2.6 g/100 g, determined by the Soxhlet extraction method [17], which is only 7% of the pork content and 19.4% of the beef content, respectively. Thus, the *Cryptotympana atrata* is one kind of delicacy with high protein and low fat (Table 2) [17]. The *Cryptotympana atrata* is also rich in amino acids, and it contains 17 kinds of amino acids, which belongs to the complete proteins, with 46.63% of all amino acids essential for the human body [16]. The content of glutamic acid, aspartic acid, phenylalanine and tyrosine are higher (Table 1) [16]. The essential amino acids contained in the food account for about 40% of the total amino acids (EAA/TAA), and the ratio of essential amino acids to non-essential amino acids (EAA/NEAA) is more than 60%, which is the most suitable proportions for the human body, according to the FAO/WHO recommendations. Therefore, the *Cryptotympana atrata* is a high quality protein source [16].

The fatty acids in the *Cryptotympana atrata* larvae mainly consist of unsaturated fatty acids [17]. Fifteen kinds of fatty acids were detected in the cicada slough of the *Cryptotympana atrata* nymphs, of which the proportion of unsaturated fatty acids was about 80% by the GC-MS analysis [18].

The Ca content and P content of the *Cryptotympana atrata* nymphs are 45.1 mg/100 g and 160 mg/100 g, respectively, measured by the methods of atomic absorption spectroscopy and spectrophotometry, which indicates that the *Cryptotympana atrata* has certain mineral nutritional values [16]. Through the analysis of trace elements in the cicada slough of the *Cryptotympana atrata*, the results showed that there were 24 trace elements in the cicada slough, including 16 essential elements for the human body, among which aluminum had the highest content of 2750 ppm [19]. The nymph of the *Cryptotympana atrata* is rich in macro and trace elements, such as K, Fe, Mn and Zn, among which the iron content is the highest, up to 60.74 mg/100 g. The iron content in the *Cryptotympana atrata* is significantly higher than that in pork, eggs, milk, grass carp and other foods [20,21]. The above results all indicate that the *Cryptotympana atrata* has a nutritional and edible value.

### 4.2. Medicinal Value and Health Care

In addition to being an edible insect as a food source, the *Cryptotympana atrata* is also widely used in medicine. There are numerous records about the medicinal value of the *Cryptotympana atrata* in the ancient books [22]. The cicada larva sheds its exoskeleton when it finally emerges as an adult, which is also called the cicada slough. The cicada slough is mainly used for treating external wind heat, cold aversion, cough, measles and poor respiration, itchy skin, sore throat and hoarseness, red and swollen eyes, children’s convulsions and night crying. It is an excellent Chinese medicine [23].

The *Cordyceps cicadae* is also widely used for medicinal purposes. The *Cordyceps cicadae* grows from the head of cicadae larvae and bifurcates from the top, like flowers; thus, it is also called the “cicadae flower” in China. Due to the sweet taste of this herbal medicine, it is the earliest medicinal cordyceps, recorded in the period of the Southern and Northern Dynasties [24]. The medicinal value of the *Cordyceps cicadae* is mainly reflected in its functions of nourishing, wind expelling and heat dissipating. As one natural health product, it also has the function of immune regulation and plays a certain role in improving kidney function. Research determined the following: cordycepin, 0.02 mg/g; adenosine, 1.9 mg/g; cordyceps polysaccharide, 94.88 mg/g; and cordycepic acid, 78.57 mg/g, in the *Cordyceps cicadae* by HPLC, 3,5-dinitrosalicylic acid colorimetry and sodium iodate colorimetry [25]. The active ingredients in the *Cordyceps cicadae* are no less than the *Cordyceps sinensis* and *Cordyceps militaris*. Moreover, modern pharmacological research shows that polysaccharide is one of the main effective components of the *Cordyceps cicadae*. With the development of modern medicine, the medicinal value of the *Cryptotympana atrata* has been gradually recognized and developed.

#### 4.2.1. Antibacterial and Anti-Inflammatory Effects

A study found that the methanol extract of the cicada slough can effectively inhibit ear swelling and hyperplasia, through experiments on contact dermatitis in mice [26]. The curative effect of the cicada slough is mediated by reducing the production of TNF-α, IFN-g and IL-6 in inflammation, which indicates that the cicada slough has the medicinal value of anti-inflammatory effect [26]. The extract of the cicada slough also has the obvious bacteriostatic effect [27]. Researchers identified that the combination of the cicada slough and *Forsythia suspensa* can enhance the antipyretic and anti-inflammatory effects by anti-free radical damage in febrile rats [28]. Zhang et al., studied the antibacterial effect of a water-soluble polysaccharide from the *Cordyceps cicadae* using the Oxford cup method and found that it had strong antibacterial activity against common pathogens [29].

#### 4.2.2. Improve Renal Function

It has been reported that the cicada slough could improve lipid metabolism and reduce albuminuria through experiments of the cicada slough on a mesangial hypertrophic nephritis rat model and identifying the corresponding impact on Toll-like receptor 4 (TLR4) expression in the renal tissue [30]. N(6)-(2-Hydroxyethyl)adenosine (HEA) was isolated from the *Cordyceps cicadae*, which was non-toxic to HK-2 cells. The study of the analysis for gene expression and western blotting revealed that HEA treatments effectively protected the human renal proximal tubule cell from DCF- and MXC-induced endoplasmic reticulum stress by regulating the pathway of GRP78/ATF6/PERK/IRE1α/CHOP [31]. It was demonstrated that cicadae polysaccharide may improve renal interstitial fibrosis in diabetic nephropathy rats with a high-fat diet treatment and an intraperitoneal injection of streptozotocin by blocking the TLR4/NF-κB and TGF-β1/Smad signaling pathways, inhibiting the inflammatory response and regulating the intestinal flora balance [32]. Researchers also analyzed and compared the effects of water extracts and ethanol extracts on the cisplatin-induced mouse kidney injury model in the treatment of an acute kidney injury, and found that both of these two extracts can reduce the renal histological changes, the production of serum creatinine and blood urea nitrogen and the levels of NO, TNF-α, IL-1β and IL-6. Moreover, water extracts were better at preventing these changes than alcohol extracts, and significantly increased the production of some peroxidase enzymes and expression levels of related proteins in kidney tissue [33].

#### 4.2.3. Anti-Oxidation and Anti-Aging Effects

The study found that cicadae polysaccharide had a strong antioxidant capacity through the determination of the total reducing power, and the scavenging ability of the DPPH radical, hydroxyl radical (OH) and superoxide anion radical [34]. By sinking water extracts of cicadae polysaccharide with different concentrations of ethyl alcohol, researchers obtained six cicadae polysaccharides CP30-CP80 and determined that CP70 had the best antioxidant activity [35]. In addition, researchers also conducted further research on the antioxidant activity of the polysaccharide component CP70 and found that it could prolong the lifespan of *Drosophila melanogaster* to a certain extent by the anti-aging activity of CP70 in vivo. Other researchers also found that the mechanism of CP70 may be related to the up-regulation of the expression of antioxidant genes *CAT*, *SOD* and MTH by a real-time PCR analysis in *Drosophila melanogaster* [36]. Another study found that the *N*-butanol site of the cicadae polysaccharide had a protective effect on glutamic acid-induced aging in PC12 cells, and the possible mechanisms of reducing the generation of oxygen free radicals in cells and preventing the occurrence of oxidative stress were also investigated [37]. Some researchers also demonstrated that the pretreatment of PC12 cells exposed to H_2_O_2_ oxidative damage with N(6)-(2-Hydroxyethyl)adenosine significantly increased cell viability and reduced reactive oxygen generation, lactate dehydrogenase release, Ca^2+^ overload and mitochondrial membrane potential collapse [38]. A new study indicated that cicadae polysaccharides of each grade had different antioxidant activities, and the P60 polysaccharide from the artificial fruiting body of the *Cordyceps cicadae* significantly increased the lifespan of *Drosophila melanogaster* by in vivo experiments [39]. 

#### 4.2.4. Enhance Immunity

Some scholars isolated acetyldopamine dimers and tetramers as well as phenolic compounds from the cicada slough [40]. Acetyldopamine is a synthetic precursor of insect melanin, which is common in insect keratins [41]. There are some crustacean components with hemostasis, anticoagulation and other functions [40]. Some researchers confirmed that the cicada slough had an anti-asthmatic effect, and its mechanism was achieved by improving the contents of IL-2 and IL-5 and alleviating chronic airway inflammation [42,43]. Another study showed that water extracts of the cicada slough could significantly inhibit the histamine release from mast cells and the occurrence of systemic allergic reactions [44].

Some researchers evaluated 13 types of nucleoside components by a HPLC/MS qualitative analysis, which were cytosine, adenine, guanine, N(6)-(2-Hydroxyethyl)adenine, deoxycytidine, hypoxanthine, uridine, deoxyinosine, deoxyuridine, inosine, guanosine, adenosine and N(6)-(2-Hydroxyethyl)adenosine, respectively. The results showed that the content of adenine, uridine, inosine, guanosine, adenosine and N(6)-(2-Hydroxyethyl)adenosine were high in the *Cordyceps cicadae* samples [45]. Adenine can promote the growth of white blood cells. Uridine is involved in the synthesis of glucuronic acid with a detoxifying effect in the liver, and improves the hypoxia tolerance of cells and the antibody level of the body [46]. Guanosine plays a neuroprotective role in an acute ischemic stroke. Adenosine has an important effect on the central nervous system and cardiovascular system.

It was indicated that the cicadae polysaccharide had a certain regulatory effect on immune functions by the intragastric administration of different doses of cicadae polysaccharide solutions in cyclophosphamide-induced immunosuppressed mice and the determination of immunological indexes [47]. Some researchers found that the cicadae polysaccharide spore powder could up-regulate IFN-γ expression and kill hepatocellular carcinoma cells by observing the therapeutic effect of spore powder on diethylnitrosamine-induced acute hepatocellular carcinoma (HCC) in mice [48]. A study demonstrated that cicadae polysaccharide components CPA-1 and CPB-1 can promote DC maturation and thus, enhance immune regulation [49]. Some researchers investigated the protective effect of the cicadae polysaccharide on an acute liver injury induced by D-galactosamine in mice and found that the protective effect was significant in a dose-dependent manner [50]. The mechanism of the protective effect may be related to the inhibition of the NF-κB inflammatory signaling pathway. The cicadae polysaccharide also exhibited the therapeutic and protective effect on carbon tetrachloride-induced hepatic fibrosis in mice by down-regulating the proportions of regulatory T cells (Tregs), thereby reducing the level of the fibrosis-promoting factor TGF-β and regulating the Th1/Th2 response balance [51].

A recent study suggested that 50% of methanol extract from the fruiting body of the *Cordyceps cicadae* could promote the proliferation of human monocytes, while the methanol extract from the insect body could inhibit the proliferation of human mononuclear cells (HMNC), indicating that the cicadae polysaccharide had a bidirectional immune regulation effect [52]. Some researchers found that the body weight, immune organ index, serum hemolysin level and secretion of related factors were increased in the immunosuppressed mice treated with the cicadae polysaccharide, which indicated that the cicadae polysaccharide had certain immunomodulatory effects [53]. It has also been reported that the artificial cultured cicada fruiting body could significantly increase the contents of serum IgA, IgG and IgM, stimulate the secretion of lymphocytes and macrophages and thus, enhance the immunomodulatory ability in H22 tumor-bearing mice [53].

#### 4.2.5. Anticonvulsive and Sedative Effects

Some researchers determined the content of 10 trace elements (Al, Cd, Cr, Cu, Fe, Mg, Mn, Ni, Se and Zn) in the cicada slough by the method of inductively coupled plasma atomic emission spectrometry [54]. Among them, the Cu, Al, Fe, Mn, Zn and Mg contents were the highest. Some studies found that the anticonvulsive effect of the cicada slough may actually function by the large amount of trace elements. It had been reported that water extracts of the cicada slough can effectively relieve carrageenan-induced hyperthermia in rats, indicating that it had anticonvulsive and sedative effects, and the mechanisms may be involved in the increase of central serotonin activity [4]. Another study investigated the anticonvulsant activities of alcohol and water extracts of the cicadae slough using the convulsive mice model induced by pentylenetetrazole (PTZ) [55]. The results showed that both alcohol and water extracts had anticonvulsive effects and that the direct inhibitory effect of water extracts was more significant than alcohol extracts. More of the contents of phosphorus and magnesium in the cicada slough may be beneficial for its clinical sedative and anticonvulsive effects [16]. In addition, the thin alcohol extracts of the *Cordyceps cicadae* could prolong the sleep time of mice, and its good analgesic effects on mice were investigated by chemical stimulation and hot plate methods [56]. The artificially-cultured *Cordyceps cicadae* significantly increased the sleep cycle and improved the sleep rate of mice, through orally giving different doses of the fruiting body and mycelium from the cicada flower, indicating that it also had good effects on improving sleep [57].

#### 4.2.6. Antitumor Effect

A study obtained 50% and 80% of cicadae polysaccharides (CP50 and CP80) from the *Cordyceps cicadae* by water extraction and alcohol precipitation methods. A further analysis showed that these two polysaccharides had inhibitory effects on the proliferation of HeLa cells, and the inhibitory effect of CP80 was more obvious than CP50 [58]. The possible mechanism was increasing the level of reactive oxygen species in HeLa cells. The cicadae polysaccharide could stimulate the expression of *Bax* and *P53* genes, and mediate apoptosis in the HeLa cell through the P53 signaling pathway [58]. Research on the toxicity of ethanol extracts (EEC) of the *Cordyceps cicadae* in the treatment for different cancer cell lines found that EEC treatment could induce the process of cell apoptosis, Ca^2+^ overload and mitochondrial membrane potential depolarization in the human gastric cancer SGC-7901 cell and increase the expression of endoplasmic reticulum stress-related proteins. The HPLC analysis showed that the EEC consisted of adenine, guanosine, adenosine and N(6)-(2-Hydroxyethyl)adenosine. These results described above suggested that the *Cordyceps cicadae* was also a potential natural product as the source of anticancer drugs [59].

#### 4.2.7. Other Medicinal Value

As one of the traditional crude drugs in East Asia, the cicada slough could significantly decrease the skin structural damage induced by irradiation [60]. A study found that the ultra-micro powder of the *Cordyceps cicadae* had an obvious anti-allergic effect by inhibiting the release of histamine in mast cells [61]. The water extracts of the cicada slough could significantly reduce the whole blood and plasma viscosity, serum triglyceride and total cholesterol levels in rats fed a high-fat diet [62]. The body weight of the rats was increased, while the blood glucose level was significantly decreased after treating diabetic rats with the cicadae polysaccharide, indicating that the cicadae polysaccharide had certain effects on reducing the blood glucose and blood lipid [63]. Another study also found that the *Cordyceps cicadae* could significantly decrease blood sugar on both normal mice and diabetic mice made by an alloxan increase of the total protein in the blood serum, the globulin content and the leukocyte count of normal rats; increase the rough endoplasmic reticulum and mitochondria of normal rats’ liver cells; and play significant roles in anti-hemorrhagic anemia and the anemia caused by the injection of phenylhydrazine hydrochloride [64].

In order to maximize the medicinal value of the *Cryptotympana atrata*, many extraction techniques are constantly being optimized and innovated. A recent study has reported a new technology, which used glutinous rice and the *Cordyceps cicadae* as the main raw materials. By adopting the strategy of the whole fermentation process, the nutritional and health-promoting components of the *Cordyceps cicadae* were fully dissolved in the rice wine. The rice wine contained most of the nutrients and physiological active substances such as polysaccharides, amino acids, cordyceps acid and trace elements in the cicada. Therefore, the *Cordyceps cicadae* can be better applied for the wine industry to take full advantage of the nutritional value and health effects in the rice wine [65].

## 5. Other Effects of the *Cryptotympana atrata*

It is quite clear that the *Cryptotympana atrata* provides many delicious food and medicinal resources for people. Moreover, the larval cicadae also help loosen the soil, and their dead carcasses can enrich the soil fertility with a lot of nitrogen, which simultaneously provides many nutrients for plant growth. However, it is necessary to consider that cicadae also have a certain ability to destroy plant health due to sucking the sap of plant roots. For the adult cicadae, the behaviors of sucking the sap from fruits and shoots, spawning and pricking with ovipositors into sawtooth branches and laying eggs in the xylem have some adverse influences, such as water loss, withered branches and even fruit pre-abscission [22]. But the amount of sap absorbed by a cicada and the adverse impact on the trees are also very small, which is hardly enough to affect the survival of trees, unless the number of cicadae is extremely large. 

Cicadae are at the bottom of the food chain, and many natural enemies including birds can prey on them. Cicadae are usually found above ground in late spring and summer, which is consistent with the period of nestlings rearing and post-fledging. Therefore, birds are probably the main biotic factor in regulating the population of cicadae [66]. Cicadae provide natural enemies with abundant food items, which contributes to the balance of the ecosystem. Moreover, on account of the relatively large quantity and variety of natural enemies, the cicada population can be better controlled within a certain range. Additionally, the cicada’s chirp has caused extensive noise pollution and severely affected people’s normal life. For example, there was a concentrated outbreak of billions of cicadae in the United States in 2004, which seriously interfered with the daily life of local residents.

## 6. Summary and Outlook

The *Cryptotympana atrata* is a common resource insect with great economic value in East and South Asia. It has high protein, low fat, low total sugar content and abundant trace elements. However, it still lacks systematical research on the development and utilization of cicadae currently. Previous studies have mostly focused on the biological characteristics of cicadae. Although some active ingredients have been identified, the research on the edible and medicinal value of cicadae is still in the preliminary stage. There is no detailed pharmacological evaluation, clinical efficacy and corresponding molecular mechanism. Therefore, more specialized research is needed for further investigation. In addition, because of the complicated extraction process, it is necessary to develop effective and efficient extraction techniques and to apply the technology to industrial production.

As the *Cryptotympana atrata* has become an increasingly popular delicacy, people’s demands for cicadae are also rising. However, in recent years, due to the environmental changes and excessive predation, the number of wild cicadae has drastically decreased, and can hardly satisfy the need of the increasing market. Therefore, a large scale of the cicada breeding industry needs to be developed. The primary challenge for artificially breeding golden cicadas is the long generation cycle and high breeding costs. The survival strategies of the cicada are not completely clear yet, and the requirements of temperature, humidity, photoperiod and artificial diets need more research for artificial breeding. Meanwhile, as a member of the insect family, the edible safety of the cicada has attracted much attention. In order to ensure the safety and reliability of its consumption, according to different feeding conditions and development stages of the cicada, it is necessary to establish a complete set of safety assessment methods for the detection of heavy metal content and fungi species, ensuring that the consumption of golden cicadas is harmless to human body.

Although the *Cryptotympana atrata* has been widely used as a medicinal insect, its active components, pharmacological evaluation and molecular mechanisms are still unclear. At present, most of the identified components are water extracts and alcohol extracts. In future studies, more appropriate separation and purification methods should be determined, based on the physical and chemical properties of active components. Possible pharmacological components and potential drug targets should be screened by conjoint analysis methods, such as network pharmacology. It could be better to identify the medicinal ingredients and study the pharmacological mechanisms through the multi-method strategy of gene overexpression or knockout, RNA interference, co-immunoprecipitation and other molecular biology technologies. Furthermore, the key active factors may also be obtained from integrated omics technology. The aim is to lay a theoretical foundation for the development and utilization of the *Cryptotympana atrata* in medicine.

This paper reviews the main research progress of the edible and medicinal value of the *Cryptotympana atrata* for the further exploitation of this insect resource in China. Currently, with the increasing demand for the cicadae, the application of the *Cryptotympana atrata* has gradually developed from a single edible medicine to healthcare products, drinks, artificial breeding and other fields. With the gradual development of the *Cryptotympana atrata* resources and the continuous improvement of its industrial chain, the practical value of this insect will continue to be investigated and utilized, which will make a great contribution to economic development in the future.

## Figures and Tables

**Table 1:** The amino acids of the *Cryptotympana atrata* in larvae and adults (g/100 g in dry weight).

Amino Acid	Larvae Content	Adult Content
Asp	6.16	6.41
Thr	2.47	2.75
Ser	2.90	2.98
Glu	7.89	8.34
Gly	3.76	3.23
Ala	4.34	4.42
Cys	0.50	0.55
Val	4.59	4.76
Met	1.58	1.52
Ile	2.80	3.03
Leu	4.27	3.18
Tyr	6.63	6.32
Phe	2.29	2.54
His	2.15	1.99
Lys	4.12	4.56
Arg	3.26	3.75
Pro	3.81	4.19

Data from reference [16].

**Table 2:** Nutrient contents of the *Cryptotympana atrata* larvae (per 100 g).

Nutrient	Content
Protein (g)	21.4
Fat (g)	2.6
Carbohydrate (g)	4.3
Calcium (mg)	133
Iron (mg)	18.7
Zinc (mg)	12.52
Retinal (μg)	9
Tocopherol (mg)	0.65

Data from reference [17].
